# A Hyaluronic Acid Based Injectable Hydrogel Formed via Photo-Crosslinking Reaction and Thermal-Induced Diels-Alder Reaction for Cartilage Tissue Engineering

**DOI:** 10.3390/polym10090949

**Published:** 2018-08-27

**Authors:** Gang Wang, Xiaodong Cao, Hua Dong, Lei Zeng, Chenxi Yu, Xiaofeng Chen

**Affiliations:** 1 Department of Biomedical Engineering, School of Materials Science and Engineering, South China University of Technology, Guangzhou 510641, China; wanggang_scut@foxmail.com (G.W.); donghua@scut.edu.cn (H.D.); happy-zl@163.com (L.Z.); eric.eu@foxmail.com (C.Y.); 2 National Engineering Research Center for Tissue Restoration and Reconstruction, Guangzhou 510006, China; 3 Key Laboratory of Biomedical Materials and Engineering, Ministry of Education, South China University of Technology, Guangzhou 510006, China

**Keywords:** injectable hydrogel, photo-crosslinking reaction, DA click chemistry, cartilage tissue engineering

## Abstract

A hyaluronic acid (HA) based injectable hydrogel with gradually increasing mechanical properties was synthesized via photo-crosslinking reaction and thermal-induced Diels-Alder (DA) reaction. The injectable hydrogel can quickly gelate within 30 s by photo-crosslinking of HA-furan under the catalysis of lithium phenyl-2,4,6-trimethylbenzoylphosphinate (LAP). This injectable property is beneficial to keep the encapsulated cell activity and convenient for clinical operation. And the mechanical properties can be control from 4.86 to 10.66 kPa by exposure time. Then, the thermal-induced DA click chemistry further occurs between furan groups and maleimide groups which gradually promoted the crosslinking density of the injectable hydrogel. The mechanical properties of the injectable hydrogel can be promoted to 21 kPa. ATDC-5 cells were successfully encapsulated in the injectable hydrogel and showed good activity. All the results suggested that the injectable hydrogel with gradually increasing mechanical properties formed by photo-crosslinking reaction and thermal-induced DA reaction has a good prospect of application in cartilage tissue engineering.

## 1. Introduction

Due to the lack of blood vessels, nerves and lymphoid tissue, articular cartilage is poor at self-repair and regeneration [1,2,3]. Thus, when the damage occurs, it is difficult for articular cartilage to repair defects by itself. Although numerous clinical treatments, including microfracture of subchondral bone, autologous chondrocyte implantation and osteoarticular transfer system, have been developed to repair articular cartilage defects, none has succeeded in regenerating articular cartilage with long-lasting [4,5,6]. With the development of regenerative medicine in the past decades, cartilage tissue engineering technique as a promising approach possesses great potential for effectively reconstructing and regenerating cartilage [7,8,9,10,11].

Hydrogel is an excellent candidate scaffold material for applications in cartilage tissue engineering due to its high water content, good viscoelastic and desirable biocompatibility [12,13]. Up to now, there are many gelation processes have been reported for formation hydrogel, such as click chemistry reactions [14,15], radical polymerization [16], Schiff base crosslinking [17,18], ionic interaction [19], hydrophobic interaction [20] and hydrogen bonding [21]. Because the advantages of high reaction yield, harmless byproducts, fast reaction kinetics, strong stereoselectivity and mild reaction conditions, the click chemistry reactions have been highly used to construct cell-compatible hydrogels over the last decade [22]. The thermal-induced Diels-Alder (DA) click chemistry reaction as classic click chemistry, which selective [4 + 2] cycloaddition between dienes and dienophiles under mild reaction conditions, has been applied to the formation of hydrogels in many studies [23,24,25]. The hydrogels which formed by thermal-induced DA click chemistry can obtain uniform network structure and have good mechanical properties, that’s very essential for cartilage tissue repair. In our previous studies, we reported multifunctional hydrogels with good elasticity, effective spatiotemporal patterning, antibacterial property and tissue adhesive ability based on thermal-induced DA click chemistry [26,27,28,29,30,31]. Nevertheless, the progress of thermal-induced DA click reaction is too slow at 37 °C, which cause hydrogels’ gelation time more than a few hours. The overlong gelation time is not conducive to the survival of cells encapsulated in hydrogels [28,32]. Meanwhile, the slow gel processing does not endow hydrogel with injectable properties. However, the injectable hydrogels are favorable in biomedical applications because their high shapes adaptability and their effective reduction of the loss of the encapsulated cells [33,34]. Usually, injectable hydrogels are formed by chemical cross-linkers [35], temperature-responsive phase transition [36], or enzymatic crosslinking reactions [30]. Most notably, injectable hydrogels formed by photo-crosslinking reactions have attracted more and more attention because the reactions can be easily controlled by UV exposure [37,38,39]. However, the too short UV exposure time go against to obtain hydrogels with strong mechanical properties and long exposure time is not conducive to the survival of the encapsulated cells. So, it is challenging but very necessary, for cartilage tissue engineering to obtain a hydrogel which gelatinization by short UV exposure time but have good mechanical properties in the same time.

In this study, we formed a hyaluronic acid based hydrogel (HA/PEG) via photo-crosslinking reaction and thermal-induced DA click chemistry reaction. The HA/PEG hydrogel can quickly gelate within 30 s by photo-crosslinking reaction of HA-Furan under the catalysis of LAP, the injectable property is beneficial to keep the encapsulated cell activity and also convenient for clinical operation. Then, thermal-induced DA click chemistry further occurred at 37 °C between furan groups and maleimide groups and the slow reaction gradually increased the mechanical properties of hydrogel. In addition, due to its good cytocompatibility, the HA/PEG hydrogel has great potential for biomedical hydrogel systems. The above characteristics make us believe that the double-crosslinking HA/PEG hydrogels can be used in cell delivery system for cartilage repair.

## 2. Materials and Methods

2,4,6-Trimethylbenzoyl chloride (TMBC) was purchased from Adamas Reagent Co., Ltd. (Shanghai, China), Dimethyl phenylphosphonite (DMPP) (98%) was purchased from Alfa Aesar (China) Chemical Co., Ltd. (Shanghai, China) Lithium bromide (LiBr) (99%) was purchased from Shanghai Aladdin Bio-Chem Technology Co., Ltd. (Shanghai, China). 2-Butanone was purchased from Hunan Hengyang Kaixin Chemical Co., Ltd. (Hengyang, China), 2-morpholinoethane sulfonic acid (MES), 4-(4,6-dimethoxy-1,3,5-triazin-2-yl)-4-methylmorpholiniumchloride (DMTMM) and 2-furylmethylamine (Furan) were purchased from Sigma-Aldrich (Guangzhou, China). Maleimide was purchased from Shanghai Macklin Biochemical Co., Ltd. (Shanghai, China). Hyaluronic acid sodium (HA, *M_w_* = 1 × 10^6^ g/mol) was purchased from Shanghai Crystal Pure Industrial Co., Ltd. (Shanghai, China). Dimaleimide poly(ethylene glycol) (Mal-PEG-Mal) (*M_w_* = 2 × 10^3^) was purchased from Shanghai Sunway Pharmaceutical Technology Co., Ltd. (Shanghai, China). Dulbecco’s Modified Eagle’s Medium (DMEM) (4.5 g/L d-Glucose), Fetal bovine serum (FBS), 0.25% Trypsin-EDTA (1X), Phosphate Buffer Saline (PBS) (pH = 7.4) and Penicillin Streptomycin (PS) were purchased from Gibco (Grand Island, NY, USA). Other reagents and solvents were of analytical grade and used as received.

### 2.1. Synthesis of LAP

**Scheme 1:**
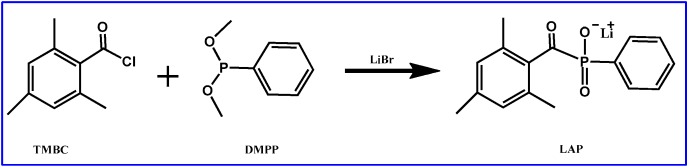
Synthesis Steps of LAP.

The lithium phenyl-2,4,6-trimethylbenzoylphosphinate (LAP), with good water solubility, polymerization rates and cell survival was prepared as originally described by Majima et al. (Scheme 1). [40]. Briefly, 3.2 g TMBC (0.018 mol) was slowly added to continuously stirred 3.0 g DMPP (0.018 mol). Then, under nitrogen atmosphere at room temperature, the mixture was stirred for 24 h. Thereafter, LiBr (6.1 g, 0.07 mol), dissolved in 2-butanone (100 mL), was added to the reaction and, subsequently, the temperature was heated to 50 °C. After the reaction duration of 10 min, the reaction was cooled to room temperature and placed for another 4 h. Subsequently, the solid precipitate in the mixture was filtered and washed 3 times with 2-butanone. The solid precipitate (LAP) was obtained by vacuum drying at room temperature for 24 h. The structure of LAP was analyzed by nuclear magnetic resonance (^1^H NMR) (BRUKER, Karlsruhe, Germany).

### 2.2. Synthesis of HA-Furan

**Scheme 2:**
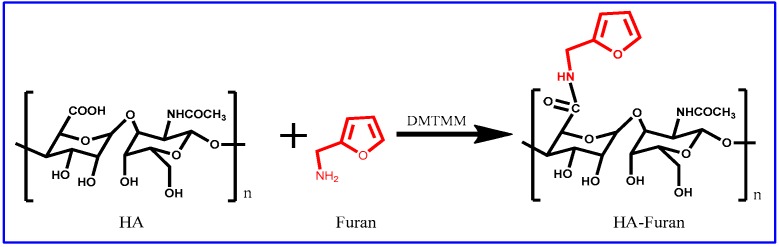
Synthesis of HA-Furan.

HA-Furan was obtained according to our previous work (Scheme 2) [28]. In brief, 0.5 g HA (1.2 mmol –COOH) was dissolved completely in MES buffer (250 mL, 100 mM), then DMTMM (1.4 g, 5 mmol) was slowly added to the mixture to activate the carboxyl groups of HA. After stirring for 1 h, Furan (220 μL, 2.4 mmol) was added dropwise and the mixture was stirred and kept at room temperature, avoiding light for 24 h. The reaction product was dialyzed (*MWCO*: 14,000 Da) against deionized water for three days and additionally lyophilized. Finally, HA-Furan as a white floccule was obtained and the structure was analyzed by ^1^H NMR (BRUKER, Karlsruhe, Germany).

### 2.3. Photo-Crosslinking Reaction of HA-Furan with LAP

**Scheme 3:**
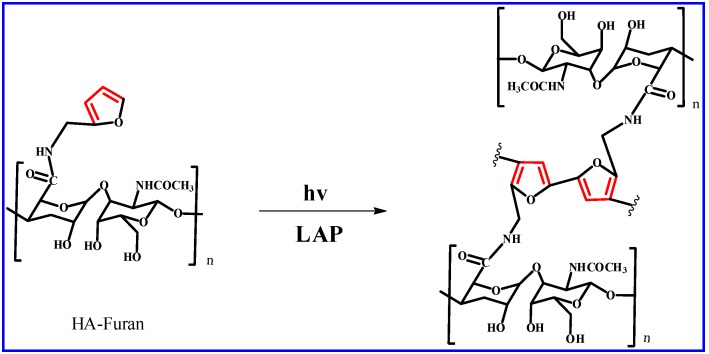
Photo-crosslinking reaction of HA-Furan with LAP.

The HA-Furan can form gels with the existence of LAP and UV (Scheme 3). The UV-Vis spectrophotometer and ^1^H NMR were used to investigate the process of photo-crosslinking of HA-Furan under the catalysis of LAP.

In the UV-Vis spectrophotometer test, HA-Furan was dissolved in deionized water to prepare dilute solution with a concentration of 300 mg/L, the solution was tested by ultraviolet visible spectrophotometer (TU-1901, Beijing Purkinje General Instrument Co., Ltd., Beijing, China) after 0 s, 30 s, 120 s and 240 s of UV irradiation. Similarly, LAP with 15 mg/L concentration was measured after 0 s, 30 s and 120 s of UV irradiation. In order to reveal the influence of UV irradiation on HA-Furan with LAP, the solution with HA-Furan (150 mg/L) and LAP (15 mg/L) was also tested by ultraviolet visible spectrophotometer Spectra analysis. The scanning range of the wavelength was from 400 to 200 nm.

In the ^1^H NMR, the HA-Furan and HA-Furan with LAP under different illumination time were performed on a Bruker AVANCE 400 (Bruker Corporation, Karlsruhe, Germany).

### 2.4. Dual-Crosslinking Reaction to form HA/PEG Hydrogel

**Scheme 4:**
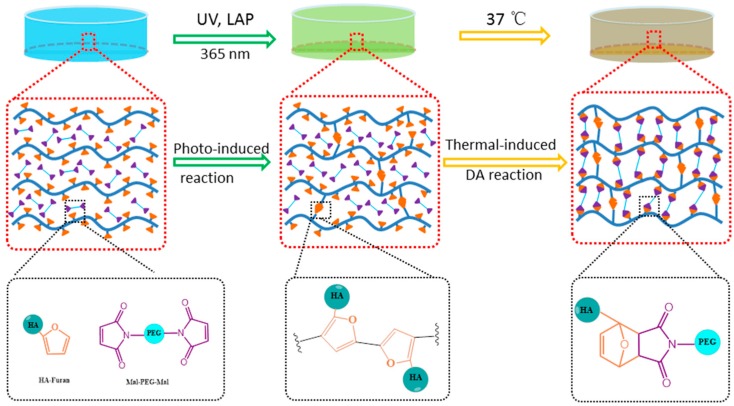
Schematic diagram of dual-crosslinking reaction to form HA/PEG hydrogel. The first step is photo-crosslinking reaction of HA-Furan under the catalysis of LAP. The second step is the thermal-induced DA reaction between Furan and Mal groups.

Lyophilized HA-Furan was totally dissolved at a concentration of 1.5% *w*/*v* in deionized water. The Mal-PEG-Mal solution with concentration of 1 g/mL (keep the mole ratio of furyl to maleimide was 1:1) was added into the solution and after stirring for 10 min, the lithium phenyl-2,4,6-trimethylbenzoylphosphin-ate (LAP) was added (the mole ratio of furyl to LAP was controlled at 1:1, 2:1 and 4:1). Then, the mixed solution was injected into a cylindrical mold (10 mm diameter). The hydrogel was formed first by way of a photo-crosslinking reaction (30 mW/cm^2^ at 365 nm). Then, as the second crosslinking process, the thermal-induced DA click chemistry will gradually occur at 37 °C for 1 day. The schematic diagram of dual-crosslinking reaction to form HA/PEG hydrogel was shown in Scheme 4.

### 2.5. The Morphology and Gelation Time of HA/PEG Hydrogel

The scanning electron microscope (SEM) (Quanta 200, FEI, Hillsboro, OR, USA) was employed to observe the morphology of the freeze-dried HA/PEG hydrogels. Before the SEM studies, the hydrogel samples were immersed in the deionized water to reach the equilibrated swelling. Then, the samples were placed in −20 °C refrigerator for 1 day. The samples were freeze-dried for 48 h. Finally, the freeze-dried samples were coated with gold in SEM coating equipment (Q 150T, Quorum Technologies Ltd., Laughton, East Sussex, UK) for 60 s for the following SEM studies.

The gelation time of the HA/PEG hydrogels were obtained using a test tube inverting method as mentioned in our previous work [28].

### 2.6. Water Absorption of HA/PEG Hydrogel

The HA/PEG hydrogels were prepared to cylinder (5 mm and diameter 10 mm) and were immersed in deionized water at room temperature until reached the equilibrium of swelling. Then, the sample was taken out from deionized water. The surface water was wiped rapidly with filter paper.

The equilibrium swelling ratio (ESR) can be calculated by the below equation:

$$\mathrm{ESR}=\frac{W_{s}-W_{d}}{W_{d}}$$

where *W_d_* and *W_s_* are the weights of the hydrogels in a freeze-dried state and an equilibrium swelling state, respectively.

### 2.7. Mechanical Testing

In order to reveal the relationship between dual-crosslinking reaction and mechanical properties of hydrogels, the stress-strain curve was tested under compression by a dynamic mechanical analyzer (DMA Q800, TA Instruments, New Castle, PA, USA) with linear ramp force 1 N/min. The hydrogel samples were prepared in cylinders with a height of 5 mm and a diameter of 10 mm.

### 2.8. Cells Encapsulation in the HA/PEG Hydrogel

Derived from mouse teratocarcinoma cells, the murine chondrocyte ATDC-5 cell line is used as a chondrogenic cell line in some studies [41]. Thus, it was chosen for encapsulation into the double-crosslinking hydrogel (HA/PEG). ATDC-5 cells were planted into the culture medium consisted of 89% DMEM, 10% FBS and 1% PS and expanded for 4–5 days in incubator with 37 °C and 5% CO_2_, the medium was updated every other day. In order to encapsulate the ATDC-5 cells, the cells were detached using 0.25% Trypsin-EDTA (1X), resuspended in the culture medium and used for the experiments. Mal-PEG-Mal and LAP solutions were dissolved with PBS and sterilized by filtration through filters with a pore size of 0.22 μm. HA-Furan powder was sterilized by Co60 irradiation sterilization. The HA-Furan solution was prepared by culture medium. ATDC-5 cells were encapsulated in the double-crosslinking hydrogels using the same procedure of gel formation. Briefly, the Mal-PEG-Mal (0.5 g/mL, 1 mL, keep the molar ratio of furan to maleimide 1:1) was first added into the 2 mL HA-Furan (1.5% *w*/*v*) solution and stirred for 10 min. Then, the suspended ATDC-5 cells (2 mL, 1 × 10^7^ cells per mL) and LAP (5%, 40 μL) were added in order. The mixed solution was injected into a cylindrical mold (10 mm diameter). The live-dead assay was used to study the cells viability encapsulated in the double-crosslinking hydrogels.

## 3. Results and Discussion

### 3.1. Chemical Structure of LAP

**Figure 1:**
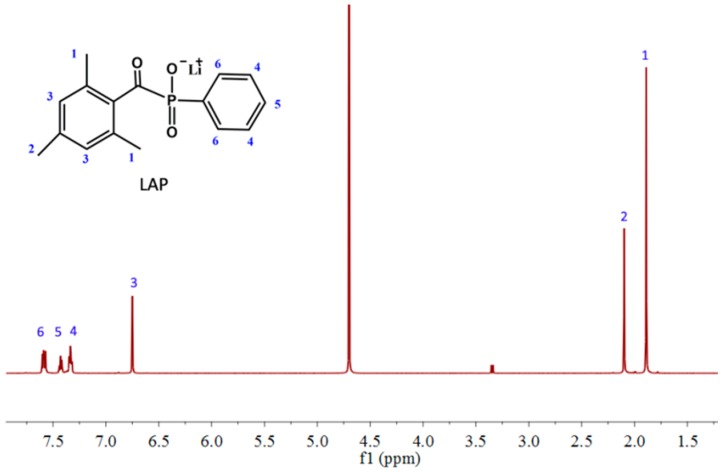
^1^H NMR spectra in D_2_O (400 MHz) of LAP showed that LAP had been successfully synthesized.

Following synthesis of the LAP (Scheme 1), ^1^H NMR was adopted to verify the structure of the LAP (Figure 1). From the Figure 1, we can observe the Peaks consistent with the proposed structure: ^1^H NMR (500 MHz, D_2_O): 7.57 (m, 2H), 7.41 (m, 1H), 7.34 (m, 2H), 6.75 (s, 2H), 2.09 (s, 3H), 1.89 (s, 6H). The result showed that the LAP was successfully synthesized.

### 3.2. Chemical Structure of HA-Furan

**Figure 2:**
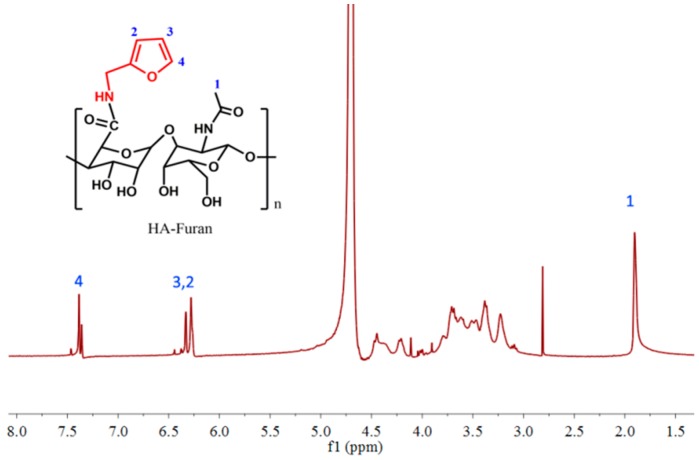
^1^H NMR spectra in D_2_O (400 MHz) of HA-Furan.

The HA-Furan was obtained by binding furfurylamine to HA using DMTMM regent (Scheme 2). The chemical structure of HA-Furan was confirmed by ^1^H NMR (Figure 2). The peaks at 6.28, 6.33 and 7.39 ppm refer to the protons of Furan, those peaks showed that the Furan group was successfully grafted to HA. The integral ratio on the ^1^H NMR spectrum suggested that HA was grafted with Furan groups on average 68%. The degree of substitution (DS) of HA-Furan was calculated as follows.

$$\mathrm{DS}=\frac{area\left(6.28\;\mathrm{ppm}\right)+area\left(6.33\;\mathrm{ppm}\right)+area\left(7.41\;\mathrm{ppm}\right)}{area\left(1.89\;\mathrm{ppm}\right)}$$

### 3.3. Photo-Crosslinking Reaction of HA-Furan with LAP

The polymerization of furan has been reported [42,43,44]. In this study, the UV-Vis spectrophotometer and ^1^H NMR were adopted to verify the process of photo-crosslinking of HA-Furan under the catalysis of LAP.

**Figure 3:**
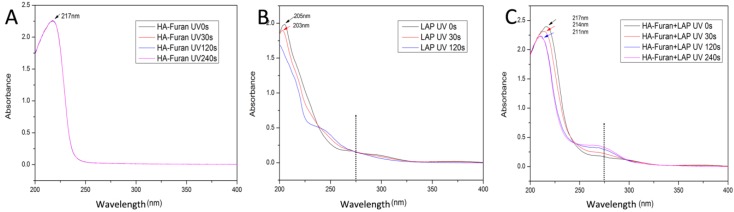
UV-Vis spectrophotometer under different illumination time. (**A**) HA-Furan; (**B**) LAP; (**C**) HA-Furan with LAP.

As shown in Figure 3A, the absorption peak of 217 nm was produced by the conjugated groups on the furan group. With the increase of irradiation time, the 217 nm peaks did not change. This indicated that UV irradiation did not change the conjugated structure of furan group. From Figure 3B, we can see that LAP without UV irradiation has an absorption peak at 205 nm. The blue shift was displayed when LAP was irradiated. When the UV irradiation was 120 s, the absorption peak of LAP was even less than 200 nm. Compared with Figure 3A,B, the absorption peak at 217 nm of the solution without UV irradiation (Figure 3C) was produced by the conjugate structure of the furan group in HA-Furan. The intensity of this peak decreased with the increased of illumination time. Meanwhile, a new band at 275 nm has appeared in Figure 3C with the increasing of illumination time. The reason for these changes is the increased conjugation structure in the gelatinization process of HA-Furan.

**Figure 4:**
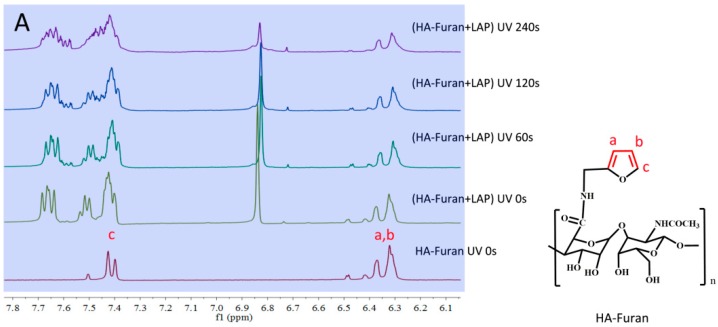

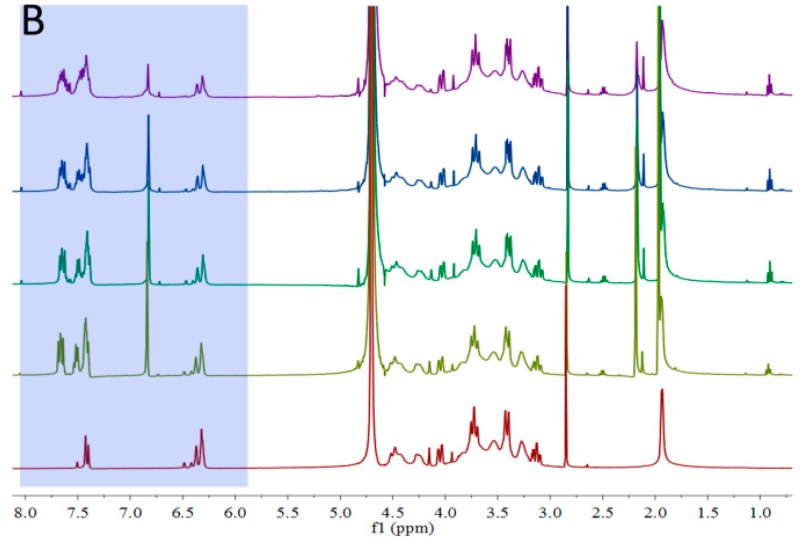
The ^1^H NMR of HA-Furan with LAP under different illumination time. (**A**) Chemical shift between 6.1 ppm and 7.8 ppm; (**B**) Chemical shift between 1.0 ppm and 8.0 ppm.

The ^1^H NMR of HA-Furan and HA-Furan with LAP under different illumination time was shown in Figure 4. Compared with the hydrogen spectrum without illumination (UV 0 s), the signal of the hydrogen spectrum on furan (a, b and c) significantly reduced with the illumination time (UV 60 s, 120 s and 240 s). This indicated that the furan group of HA-Furan constantly participated in the process of hydrogel formation with the continuous illumination. Combined with the results of UV-Vis spectrophotometer in Figure 3, we can know that the furan group of HA-Furan took part in the process of hydrogel formation reaction with the existence of LAP and UV but the conjugate double bond of furan was not destroyed and the conjugate effect of the system was enhanced. So, we believe the gelation of HA-Furan is more likely caused by polymerization of furan under the catalysis of LAP as the initiator which was shown in Scheme 3.

### 3.4. Gelation Time of Double-Crosslinking Hydrogel (HA/PEG)

**Figure 5:**
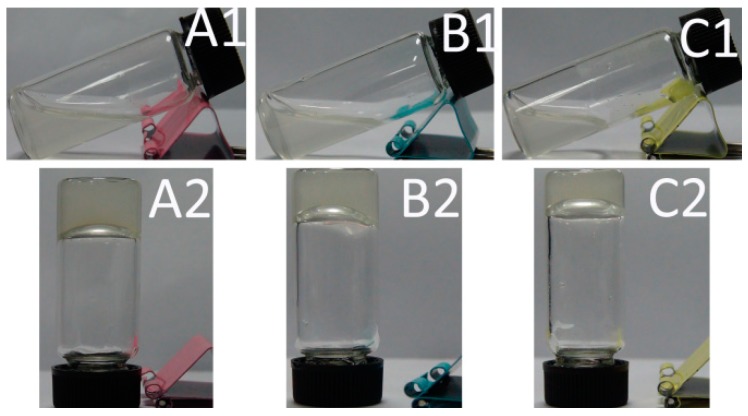
Gelation photos of HA/PEG hydrogels. (**A1**–**C1**) were the photos before gelation of HA/PEG-I, HA/PEG-II, HA/PEG-III, respectively; (**A2**–**C2**) were the photos after gelation of HA/PEG-I, HA/PEG-II, HA/PEG-III, respectively.

The gelation time (GT) of double-crosslinking hydrogel relied on photo-crosslinking reaction. Table 1 shows the gelation time on the concentration of LAP. Keeping a constant molar ratio of Furan and Mal, the gelation time increased with the decreased LAP concentration (Figure 5 and Table 1). The start gelling time of the HA/PEG can easily control by UV light and the hydrogel can rapid formation within 30 s. This rapid controllable gelation behavior is not only beneficial for cell encapsulation and survival but also beneficial for the operation of the HA/PEG hydrogel in clinics.

### 3.5. Morphology of HA/PEG Hydrogel

**Figure 6:**
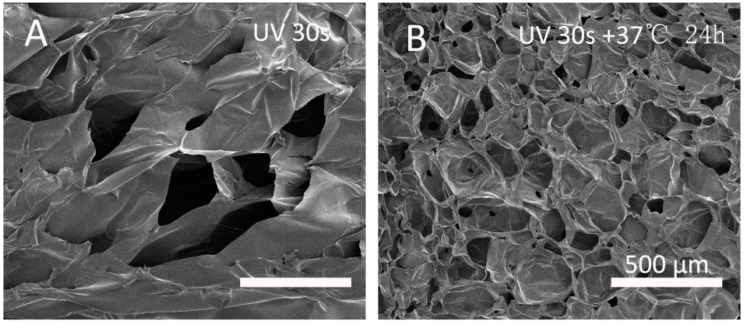
SEM of HA/PEG-III hydrogel formed via photo-crosslinking reaction and thermal-induced DA reaction. (**A**) only UV light 30 s; (**B**) UV 30 S, then 37 °C further reaction for 24 h. Scale bar: 500 μm.

The relationship between the dual-crosslinking reaction and the network structure can be revealed by the morphology of HA/PEG-III hydrogel (Figure 6). It was found that the pore size of freeze dried hydrogel formed only by photo-crosslinking reaction (Figure 6A, only UV light 30 s) is much bigger than that formed by double-crosslinking reaction (Figure 6B, UV 30 s, then 37 °C further reaction for 24 h). It is well known that the pore size of hydrogels is attributed to its crosslinking density. Therefore, the dual-crosslinking reaction can increase the crosslinking density of the HA/PEG hydrogel.

### 3.6. Mechanical Properties of HA/PEG Hydrogel

**Figure 7:**
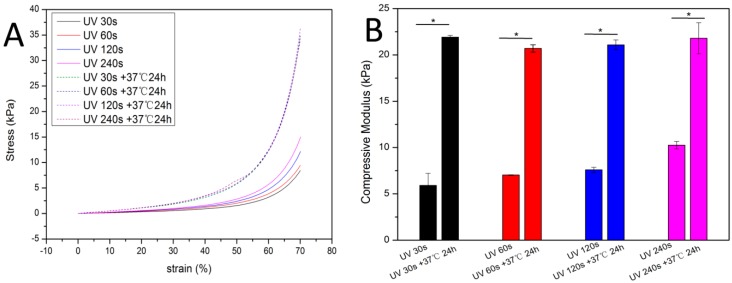
The mechanical properties of HA/PEG-III. (**A**) Typical compressive stress–strain curves; (**B**) The compressive modulus of the hydrogels. Error bars represent the SD of measurements performed on 3 samples.

In order to reveal the relationship between dual-crosslinking reaction and mechanical properties, HA/PEG-III hydrogel was tested under compression by a dynamic mechanical analyzer (DMA Q800 USA). From the resulting compressive stress-strain plots in Figure 7A, the mechanical properties of the HA/PEG-III hydrogel increased with the increasing of the exposure time from 30 s to 240 s. That was because the photo-crosslinking reaction was the first crosslinking reaction, the crosslinking density depended the exposure time of UV. Then, the light forming hydrogel was placed under a temperature of 37 °C for 24 h, the thermal-induced Diels-Alder (DA) click chemistry, as the second crosslinking process, slowly occurred to further improve the crosslinking density of the HA/PEG-III. To our surprise, the mechanical properties of the HA/PEG-III hydrogels with different exposure time tend to be same after placed under 37 °C for 24 h. This result indicated that the final mechanical properties of the hydrogels depend on the concentration of Furan groups and Mal groups. The compressive modulus of the hydrogels (Figure 7B) was calculated from the stress-strain curves and shown the same trend. Therefore, the injectable HA/PEG hydrogel with rapid gelation (30 s) and gradually increasing mechanical properties can be obtained.

### 3.7. Water Absorption

**Figure 8:**
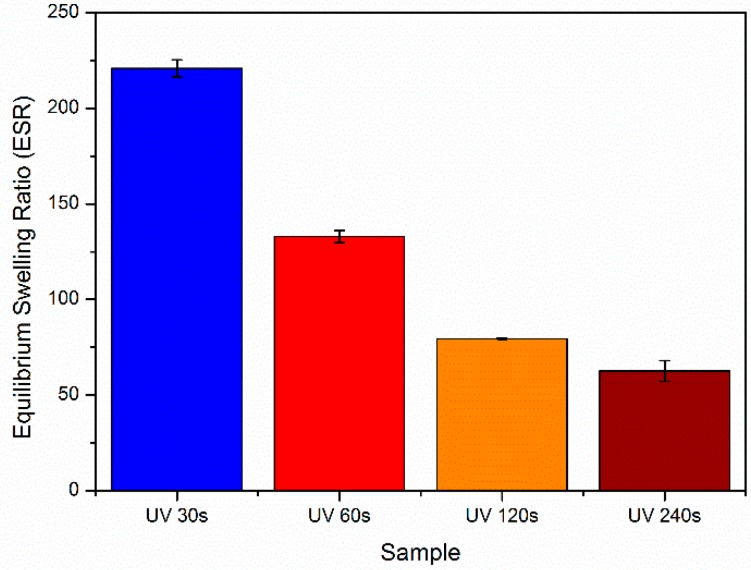
The equilibrium swelling ratio of HA/PEG hydrogels with different UV exposure time. Error bars represent the SD of measurements performed on 4 samples.

The equilibrium swelling ratio (ESR) is a useful parameter for hydrogels, to be used in biomaterials. As shown in Figure 8, the ESR of HA/PEG hydrogel decreased with UV exposure time increased. This behavior could be attributed to further progress of photo-crosslinking reaction along with the increase of exposure time.

### 3.8. Cell Viability in the Hydrogel

**Figure 9:**
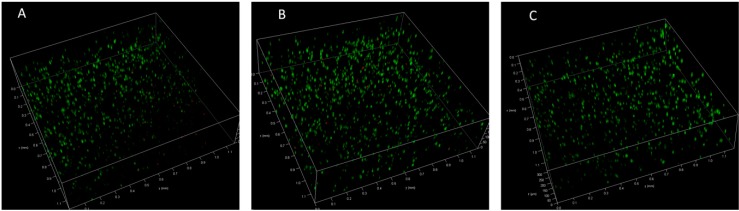
Live-dead assay of encapsulated ATDC-5 cells in HA/PEG hydrogel (**A**) 1 day; (**B**) 4 days; (**C**) 7 days. (Living cells appearing green and dead cells appearing red).

The live-dead assay was using to evaluate the viability of encapsulated ATDC-5 cells in HA/PEG-III hydrogel after cultured 7 days (Living cells and dead cells appearing green and red, respectively). As shown in Figure 9, the high level of cell survival after 7 days culture in hydrogel suggested that the injectable HA/PEG-III hydrogel formed via a photo-crosslinking reaction and thermal-induced DA click chemistry has good cellular compatibility.

## 4. Conclusions

A dual-crosslinking reaction that formed HA/PEG hydrogel with injectable properties, gradually increasing mechanical properties and good cell compatibility has been demonstrated in this study. The injectable HA/PEG hydrogel can quickly gelate within 30 s by way of a photo-crosslinking reaction of HA-Furan with LAP, which is beneficial for keeping the encapsulated cell activity and convenient for clinical operation. The thermal-induced DA click chemistry further slowly occurred between Furan groups and Mal groups, which gradually increased the crosslinking density and mechanical properties of the hydrogel. The injectable HA/PEG hydrogel successfully encapsulated the ATDC-5 cells in situ and showed good cytocompatibility. The above results reveal that the injectable hydrogel with gradually increasing mechanical properties, formed via a photo-crosslinking reaction and thermal-induced DA click chemistry, has great potential applications in cartilage tissue engineering.

## Figures and Tables

**Table 1:** The gelation time (GT) of HA/PEG hydrogel.

Sample	[Furan]:[Mal]:[LAP]	GT (s)
HA/PEG-I	1:1:1	20
HA/PEG-II	2:2:1	25
HA/PEG-III	4:4:1	30
